# Correlation between weight-adjusted-waist index and hypertension in the US population: based on data from NHANES 2005–2018

**DOI:** 10.3389/fcvm.2024.1416836

**Published:** 2024-11-12

**Authors:** Anwu Huang, Bin Lin, Zhuyin Jia, Xiaojun Ji, Yalong Chen

**Affiliations:** Department of Cardiology, Wenzhou Central Hospital, Wenzhou, Zhejiang, China

**Keywords:** NHANES, weight-adjusted-waist index, hypertension, obesity, cross-sectional study

## Abstract

**Objectives:**

This study aimed to investigate the association between the weight-adjusted waist index (WWI) and the prevalence of hypertension in U.S. adults.

**Methods:**

Data were sourced from the National Health and Nutrition Examination Survey (NHANES) spanning 2005–2018. In our cross-sectional study, we focused on the non-institutional U.S. population over the age of 18 from various communities in the United States. WWI is derived by dividing waist circumference by the square root of body weight. The definition of hypertension was based on self-reported history of hypertension, antihypertensive drug use, and blood pressure measurements. Participants without complete information on WWI and hypertension were excluded. The independent relationship and consistency between WWI and hypertension were assessed through weighted multivariate regression. The Pearson correlation test was used to detect the association between WWI and BMI. Subgroup analyses were used to verify the stability of the relationship between WWI and the prevalence of hypertension, and interaction tests were also conducted by gender, age, smoking, and triglycerides.

**Results:**

Among the 37,299 participants included, the hypertension prevalence was 33.9%. After adjusting for confounding variables, WWI demonstrated a significant association with hypertension. Individuals in the top quarter of WWI had a 2.27fold higher chance of hypertension prevalence compared with the bottom quarter (OR = 2.27, 95% CI 1.97–2.61; *P* < 0.0001). Subgroup analysis highlighted that this association was particularly pronounced in males aged ≤60 years.

**Conclusion:**

The findings underscore a robust correlation between elevated WWI and a heightened risk of hypertension, especially in males aged ≤60 years.

## 1 Introduction

Hypertension, a prevalent contributor to heart disease (1), threatens the well-being of numerous individuals and places a significant financial strain on the global economy (2–4). Research indicates that each 10mmHg increase in systolic blood pressure is associated with a 45% elevated risk of coronary artery disease and a 65% higher probability of stroke (5). Globally, over half of cardiovascular-related deaths are attributed to high blood pressure and its complications (6, 7). With the aging population and the prevalence of adverse lifestyle factors, the number of individuals with hypertension continues to rise. From 1990 to 2019, the prevalence of hypertension has nearly doubled (8), highlighting the urgency of identifying modifiable risk factors.

The occurrence of hypertension is related to many factors. In previous studies, the aging of the population, smoking, gender and other factors have affected the incidence of hypertension (9, 10). Interestingly, one recent study showed that triglycerides had a stronger relationship with blood pressure than other lipid markers (11), and another meta-study found that people who lost significant amounts of weight also had lower blood triglycerides (12). This leads us to think about the relationship between obesity and hypertension.

Prior research has indicated a strong connection between obesity and hypertension onset (13–16). For every kilogram of weight lost, systolic blood pressure will drop by about 1 mmHg (17). In obese patients with hypertension, bariatric surgery can significantly reduce weight and improve blood pressure (18). Present evaluations of obesity primarily concentrate on conventional measurements of the body, with body mass index (BMI) being the most frequently utilized. However, BMI does not differentiate between fat and lean tissue (19), and in some studies, BMI has been found maybe inadequate for predicting cardiovascular events (20). In response, Park et al. proposed to use WWI as a new indicator to evaluate obesity (21). Previous studies have shown that WWI is more associated with elevated ferritin and the development of diabetes than BMI (22, 23), which means WWI to assess obesity can better reflect the situation of central obesity. Numerous investigations have demonstrated that WWI can predict the likelihood of cardiovascular events (24–26) and WWI can predict mortality in patients with metabolic syndrome (27). However, there are only two studies on the relationship between WWI and hypertension. The Rural Chinese Cohort Study (28) explored the relationship between WWI and hypertension in rural adults aged over 18, identifying a positive correlation between WWI and hypertension onset. However, among individuals aged over 60, no statistically significant association was found. In contrast, another study by Wang et al. (29), based on NHANES data of Americans over 60, demonstrated a positive association between WWI and hypertension. The results of these studies are inconsistent.

To address this gap and verify whether the relationship between WWI and the incidence of hypertension is more predictive in specific populations and ages, this study extended the age range and increased the sample size using data from the NHANES database to delve deeper into the link between WWI and hypertension. Subgroup analyses were used to verify the stability of the relationship between WWI and the prevalence of hypertension. The ultimate objective is to reduce hypertension rates through targeted interventions based on WWI.

## 2 Materials and method

### 2.1 Survey description

Data for this study were sourced from NHANES, a national survey conducted by the National Center for Health Statistics (NCHS) to evaluate health and nutrition across the U.S. population through a complex multistage probability design. NHANES research protocols were approved by the NCHS Research Ethics Review Committee, and all participants provided written informed consent. NHANES data in the research can be found on the website at https://www.cdc.gov/nchs/nhanes/.

### 2.2 Participant

**Figure 1:**
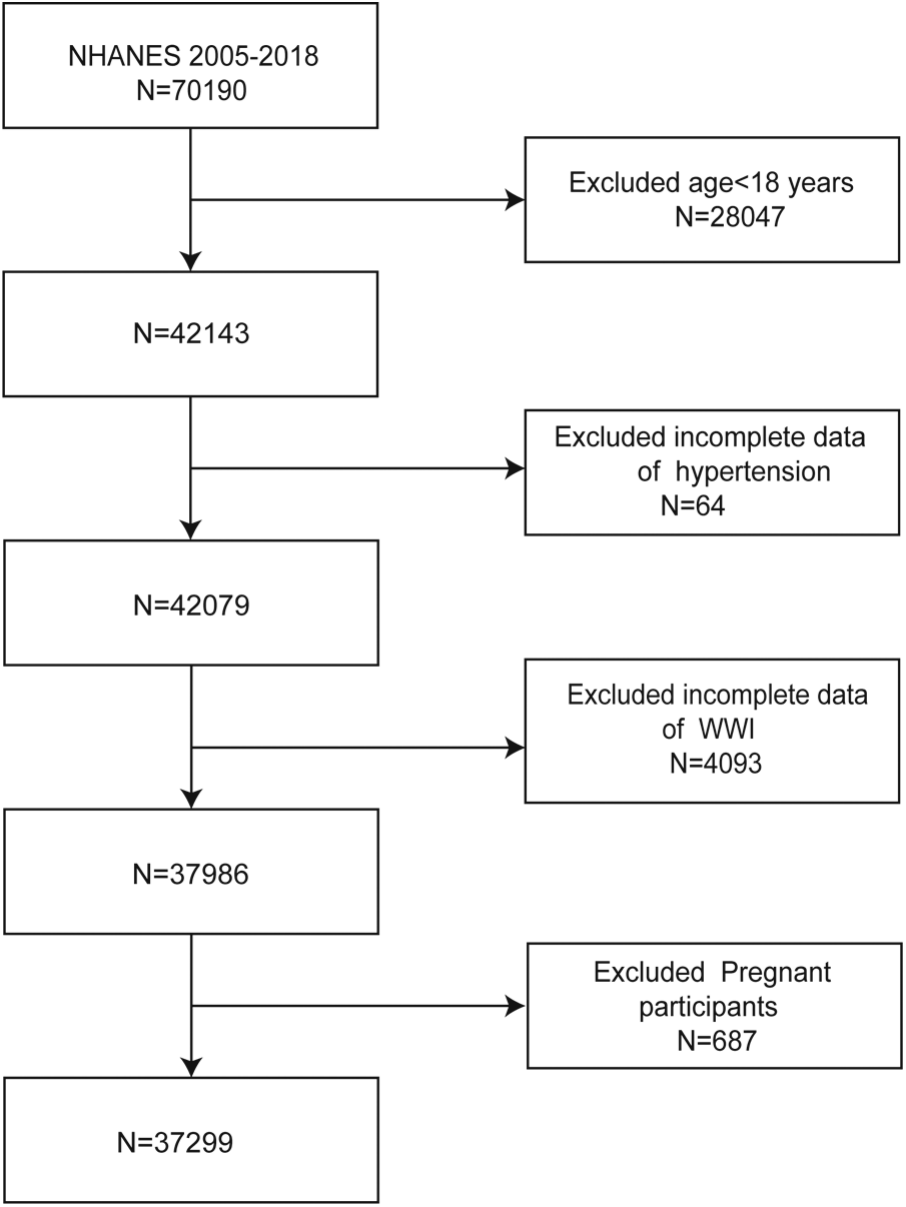
Flowchart of the sample selection from NHANES 2005–2018.

To investigate the potential relationship between WWI and hypertension, data were extracted from seven NHANES survey cycles spanning 2005–2018. Participants lacking complete WWI or hypertension data were excluded from the analysis. Initially, 70,190 individuals were included; however, after excluding those under 18 years of age (*n* = 28,047), those missing hypertension data (*n* = 64), WWI data (*n* = 4,093), or pregnant (*n* = 687), the final sample consisted of 37,299 eligible participants (Figure 1).

### 2.3 Weight-adjusted waist circumference index

WWI, a novel indicator of central obesity proposed by Park (21), is derived by dividing waist circumference by the square root of body weight. In this study, WWI served as the independent variable, with higher WWI values indicating greater central obesity.

### 2.4 Hypertension

All individuals had their blood pressure measured at a mobile examination center (MEC).After sitting and resting for at least 5 min, each participant's blood pressure was measured three times by a clinician. The participant's blood pressure was recorded as the average of the three times.

People who met one of the following criteria are considered to have hypertension: (1) The answer to the problem “Have you ever been told that you had hypertension?” was yes; (2) Taking antihypertensive medication; (3) The participants’ average systolic or diastolic blood pressure exceeded the standard range across three blood pressure readings.

### 2.5 Covariates

In accordance with previous literature, several covariates were selected to control for confounding factors in the analysis of the WWI-hypertension relationship. These included demographic variables such as age (≥18 years), gender (female/male), race (Mexican American/Non-Hispanic White/Non-Hispanic Black/Other Hispanic/Other races), education level (Above high school/High school or general education development/Less than high school/Others), and smoking status (defined as having smoked ≥100 cigarettes in one's lifetime). Additionally, health-related measurements such as fasting plasma glucose (µIU/mL), hemoglobin A1c (%), serum creatinine (µmol/L), serum uric acid (µmol/L), total cholesterol (mmol/L), high-density lipoprotein cholesterol (mmol/L), low-density lipoprotein cholesterol (mmol/L), triglycerides (mmol/L), alanine transaminase (U/L), aspartate transaminase (U/L), waist circumference (cm), and weight (kg) were included.

### 2.6 Statistical analysis

Statistical analyses were conducted in accordance with NCHS guidelines, incorporating sample weights and accounting for the complex multi-stage cluster survey design. Continuous variables were presented as mean ± standard deviation (SD), while categorical variables were expressed as percentages. Differences between WWI groups were evaluated using the weighted *t*-test for continuous variables and the weighted Chi-square test for categorical variables. The Pearson correlation test was used to detect the association between WWI and BMI. A multivariate logistic regression model was employed to explore the potential association between WWI and hypertension. Missing values of categorical variables are included in multiple regression as a separate group, and missing values of continuous variables are included in multiple regression after being filled with the mean or median. Three models were constructed: Model 1 without any covariate adjustments, Model 2 adjusted for gender, race, and age, and Model 3 further adjusted for gender, age, race, education level, smoking status, fasting plasma glucose, hemoglobin A1c, serum creatinine, serum uric acid, total cholesterol, high-density lipoprotein cholesterol, low-density lipoprotein cholesterol, triglycerides, alanine transaminase, and aspartate transaminase. Subgroup analyses were also performed based on gender, age, smoking status, and triglyceride levels. All statistical analyses were conducted using R and EmpowerStats, with a two-sided *P*-value of less than 0.05 considered statistically significant.

## 3 Results

### 3.1 Baseline characteristics of participants

The study included 37,299 participants with a mean age of 47.76 ± 13.70 years, comprising 49.70% men and 50.30% women. WWI was categorized into four quartiles: 7.72–10.41, 10.41–11.01, 11.01–11.60, and 11.60–15.70. Hypertension was prevalent in 33.9% of the participants, with the prevalence of hypertension rising as WWI increased (quartile 1: 11.98%, quartile 2: 26.12%, quartile 3: 37.47%, quartile 4: 52.35%; *P* < 0.0001) (Table 1).

**Table 1:** Baseline characteristics of study population according to weight-adjusted-waist index tertiles, weighted.

Baseline characteristics	Weight-adjusted waist index					*P*-value
	Overall	Q1 (7.72–10.41)	Q2 (10.41–11.01)	Q3 (11.01–11.60)	Q4 (11.60–15.70)	
No. of participants	37,299	9,325	9,324	9,325	9,325	
Age (years)	47.76 ± 13.70	35.22 ± 13.70	44.67 ± 15.02	51.04 ± 16.01	57.35 ± 16.46	<0.0001
Gender (%)						<0.0001
Male	49.70	58.33	52.95	47.86	34.21	
Female	50.30	41.67	47.05	52.14	65.79	
Race (%)						<0.0001
Mexican American	16.10	5.38	8.95	10.54	10.33	
Other Hispanic	9.66	4.82	5.61	6.42	5.79	
Non-Hispanic White	40.96	66.47	66.51	65.50	68.75	
Non-Hispanic Black	21.93	15.19	10.34	9.76	9.11	
Other Races	11.34	8.15	8.59	7.78	6.03	
Education level (%)						<0.0001
Less than high school	23.29	10.42	13.53	18.57	22.31	
High school or GED	21.63	19.31	22.14	25.33	27.05	
Above high school	49.2	70.21	64.28	56.05	50.58	
Others	5.88	0.06	0.05	0.06	0.05	
Smoking status (%)						<0.0001
Smoker	42.38	38.45	44.15	48.06	48.70	
Non-smoker	57.62	61.55	55.85	51.94	51.30	
Hypertension (%)	33.9	11.98	26.12	37.47	52.35	<0.0001
Systolic blood pressure (mmHg)	123.36 ± 17.67	116.38 ± 13.10	120.50 ± 15.25	124.65 ± 16.89	128.38 ± 18.30	<0.0001
Diastolic blood pressure (mmHg)	69.96 ± 12.23	68.96 ± 10.43	71.71 ± 11.14	71.64 ± 11.77	69.82 ± 12.95	<0.0001
Body mass index (kg/m^2^)	28.90 ± 6.83	24.51 ± 4.56	27.86 ± 5.30	30.35 ± 6.10	33.79 ± 7.60	<0.0001
Fasting plasma glucose (µIU/mL)	6.06 ± 1.99	5.38 ± 1.07	5.75 ± 1.51	6.06 ± 1.79	6.58 ± 2.23	<0.0001
Hemoglobin A1c (%)	5.73 ± 1.07	5.27 ± 0.55	5.49 ± 0.79	5.71 ± 0.95	6.02 ± 1.14	<0.0001
Serum creatinine (µmol/L)	79.67 ± 36.78	79.56 ± 26.07	77.82 ± 26.82	78.10 ± 30.46	78.19 ± 33.44	0.0001
Serum uric acid (µmol/L)	323.94 ± 84.95	306.62 ± 77.30	320.23 ± 82.27	328.17 ± 84.12	337.95 ± 85.46	<0.0001
Total cholesterol (mmol/L)	4.95 ± 1.08	4.73 ± 0.99	5.09 ± 1.02	5.13 ± 1.09	5.04 ± 1.14	<0.0001
HDL-C (mmol/L)	1.37 ± 0.41	1.48 ± 0.43	1.39 ± 0.43	1.33 ± 0.41	1.31 ± 0.39	<0.0001
LDL-C (mmol/L)	2.91 ± 0.92	2.74 ± 0.85	3.01 ± 0.88	3.05 ± 0.94	2.93 ± 0.96	<0.0001
Triglycerides (mmol/L)	1.41 ± 1.24	1.09 ± 0.97	1.40 ± 1.14	1.56 ± 1.33	1.65 ± 1.24	<0.0001
ALT (U/L)	25.06 ± 20.53	22.79 ± 14.22	26.05 ± 19.63	26.86 ± 18.73	25.53 ± 23.61	<0.0001
AST (U/L)	25.46 ± 16.93	24.77 ± 13.47	25.16 ± 14.30	25.74 ± 16.09	25.40 ± 16.71	0.0002
Waist circumference (cm)	98.51 ± 16.59	84.48 ± 10.35	95.72 ± 11.55	103.85 ± 12.82	114.38 ± 15.94	<0.0001
Weight (kg)	81.08 ± 21.30	73.46 ± 16.60	80.93 ± 19.21	85.89 ± 21.17	91.06 ± 24.91	<0.0001

GED, general educational development; HDL-C, high-density lipoprotein cholesterol; LDL-C, low-density lipoprotein cholesterol; ALT, alanine transaminase; AST, aspartate transaminase.

### 3.2 The correlation of waist-to-weight ratio with body mass index

In Table 1, the average BMI of the study population reached 28.90, and the BMI gradually increased with the increase of WWI (quartile 1: 24.51 ± 4.56, quartile 2: 27.86 ± 5.30, quartile 3: 30.35 ± 6.10, quartile 4: 33.79 ± 7.60; *P* < 0.0001). Supplementary Table S1 shows this positive correlation between WWI and BMI persisted even after stratified by age, sex, and smoking group.

### 3.3 The correlation of waist-to-weight ratio and body mass index with hypertension

Table 2 illustrates the correlation between WWI and hypertension prevalence. The findings demonstrate a strong association between higher WWI and an increased likelihood of hypertension. In both the unadjusted and fully adjusted models, WWI was positively correlated with hypertension prevalence. As a continuous variable, each one-unit increase in WWI was associated with a 42% higher risk of having hypertension after full adjustment (Model 3 OR = 1.42, 95% CI 1.34–1.51). This relationship remained significant even when WWI was analyzed by quartiles, with participants in the highest quartile showing a 127% greater risk compared to those in the lowest quartile (OR = 2.27, 95% CI 1.97–2.61; *P* for trend <0.0001). Supplementary Table S2 shows an association between higher BMI and an increased likelihood of hypertension, each one-unit increase in BMI was associated with a 6% higher risk of having hypertension after full adjustment (Model 3 OR = 1.06, 95% CI 1.06–1.07).

**Table 2:** Association between weight-adjusted-waist index and the prevalence of hypertension.

Weight-adjusted-waist index	Model 1		Model 2		Model 3	
	OR (95% CI)	*P*-value	OR (95% CI)	*P*-value	OR (95% CI)	*P*-value
Continuous	2.43 (2.36, 2.51)	<0.0001	1.74 (1.68, 1.80)	<0.0001	1.42 (1.34, 1.51)	<0.0001
Categories						
Quartile 1	1.0 (Ref)		1.0 (Ref)		1.0 (Ref)	
Quartile 2	2.51 (2.32, 2.70)	<0.0001	1.78 (1.64, 1.94)	<0.0001	1.47 (1.30, 1.67)	<0.0001
Quartile 3	4.33 (4.03, 4.65)	<0.0001	2.41 (2.22, 2.61)	<0.0001	1.83 (1.60, 2.08)	<0.0001
Quartile 4	7.76 (7.22, 8.35)	<0.0001	3.47 (3.19, 3.79)	<0.0001	2.27 (1.97, 2.61)	<0.0001
*P* for trend	<0.0001		<0.0001		<0.0001	

Model 1: Unadjusted; Model 2: Adjusted for gender, age, race at baseline; Model 3: Adjusted for gender, age, race, education level, smoking status, Fasting plasma glucose, Hemoglobin A1c, Serum creatinine, Serum uric acid, Total cholesterol, fasting plasma glucose, total cholesterol, high-density lipoprotein cholesterol, low-density lipoprotein cholesterol, triglyceride, alanine transaminase, aspartate transaminase at baseline.

### 3.4 Subgroup analysis

**Figure 2:**
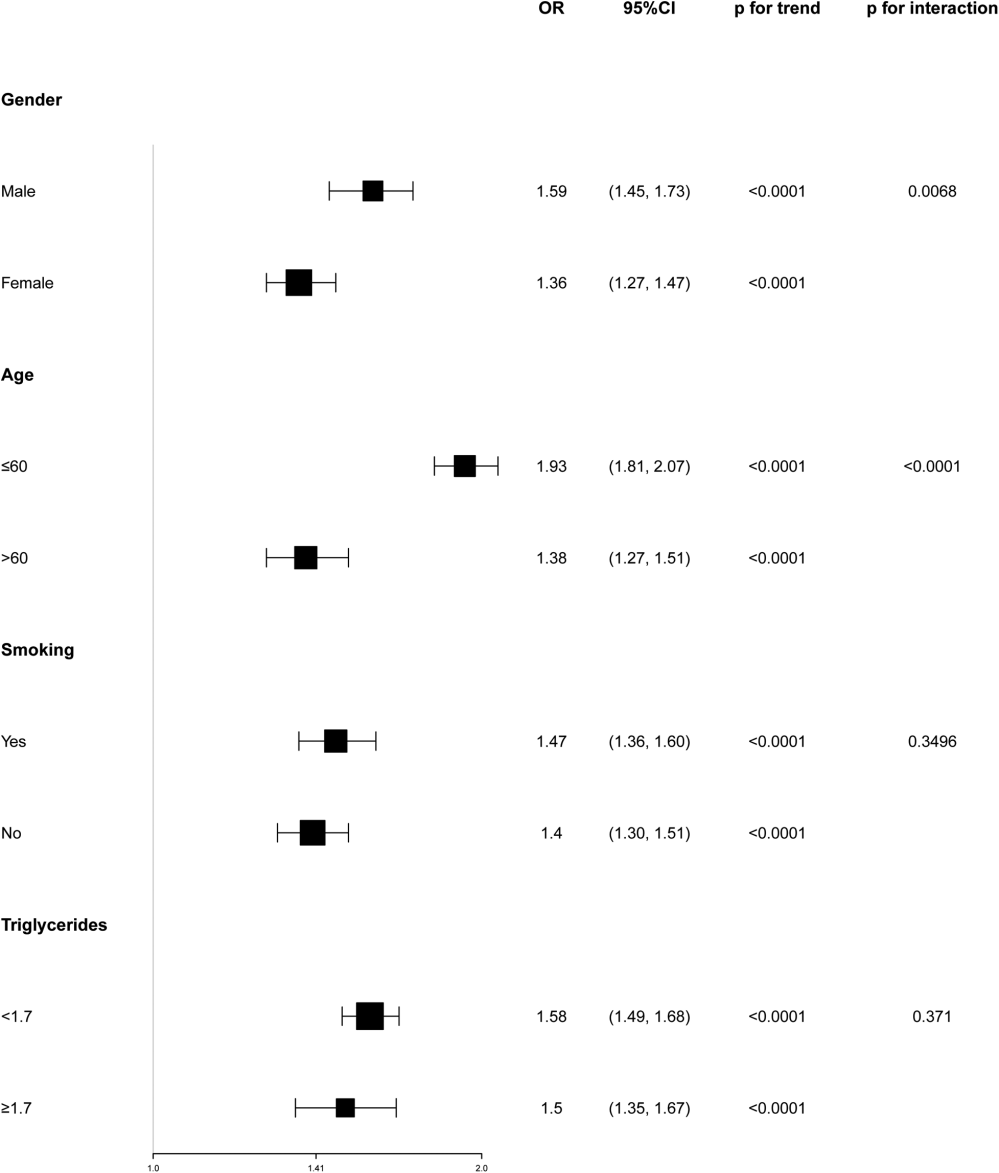
Subgroup analyses of the association between weight-adjusted waist index and the prevalence of hypertension.

To evaluate the consistency of the WWI-hypertension association across various population groups, subgroup analyses and interaction tests were conducted based on age, sex, smoking, and triglycerides. The results depicted in Figure 2 revealed a positive correlation between WWI and hypertension prevalence in all subgroup analyses. Notably, a more pronounced correlation between WWI and high blood pressure prevalence was observed in gender and age stratification (*P* for interaction <0.05). Conversely, no significant association was found between WWI and high blood pressure prevalence in smoking and triglycerides subgroups (*P* for interaction >0.05).

## 4 Discussion

This study assessed the relationship between WWI and the incidence of hypertension among American adults. Our findings indicate that greater levels of WWI are linked to an enhanced likelihood of hypertension in adults. After accounting for relevant factors, the highest WWI level (11.60–15.70) was correlated with a 2.27 times greater risk of hypertension compared to the lowest level (7.72–10.41). Moreover, WWI was also more associated with the incidence of hypertension than BMI. Subgroup analyses revealed a stronger positive correlation between WWI and hypertension prevalence in men aged 60 or younger. As the first study to investigate the link between WWI and hypertension in the U.S. adult population, it underscores important gender- and age-related differences in this relationship. The findings suggest that WWI is a meaningful predictor of hypertension, especially in younger males, and that addressing obesity through WWI measurement could be an effective strategy for reducing hypertension rates.

Historically, BMI has been the most widely used anthropometric measure for assessing obesity. Numerous studies have established a clear relationship between BMI and hypertension. For example, Hollie et al., utilizing data from the Health and Retirement Study (30), found a strong association between obesity and high blood pressure. Males with a BMI of 28 kg/m^2^ or higher had a 2.3-fold increased risk of developing hypertension compared to those with a BMI below 28 kg/m^2^. Similarly, women with a BMI between 24 and 28 kg/m^2^ or a BMI of 28 kg/m^2^ or higher had 1.7 and 2.4 times greater risk, respectively, of developing hypertension compared to those with a healthy BMI. Additionally, the Longitudinal Aging Study (31) reported that older adults who were overweight or obese had significantly higher rates of hypertension. It also highlighted that individuals with an elevated waist circumference and waist-to-hip ratio had a 1.16 and 1.42 times greater risk of developing hypertension, respectively, underscoring the importance of central obesity. Recent developments in obesity pharmacotherapy have introduced treatments that may influence blood pressure. For instance, the SURMOUNT-1 trial, which involved obese or overweight patients with weight-related complications, demonstrated that 72 weeks of tirzepatide treatment led to significant reductions in both weight and blood pressure. A *post hoc* analysis suggested that blood pressure reduction was closely associated with weight loss (32). However, BMI, as a basic measure of body fat, does not distinguish between fat and lean tissue. Research has shown that individuals with normal BMI can have a higher mortality rate than those with obese BMI due to differences in fat distribution (33), indicating the need for updated anthropometric measures to assess central obesity. In this context, WWI has emerged as a more accurate predictor of central obesity. A prospective study from the Zhejiang Metabolic Syndrome Cohort found WWI to be significantly more predictive of coronary heart disease and non-accidental death compared to traditional measures like BMI, waist circumference, and waist-to-hip ratio (34). As a result, WWI, a novel metric for central obesity, was selected to investigate its association with hypertension prevalence.

Two studies have previously examined the link between WWI and hypertension. The Rural Chinese Cohort Study (28) explored the relationship between WWI and hypertension in rural adults aged over 18, identifying a positive correlation between WWI and hypertension onset. However, among individuals aged over 60, no statistically significant association was found. In contrast, another study by Wang et al. (29), based on NHANES data of Americans over 60, demonstrated a positive association between WWI and hypertension. These conflicting findings prompted us to expand both the sample size and the age range to provide a more comprehensive analysis of the relationship between WWI and hypertension. Our research identified a significant association between WWI and hypertension prevalence in American adults aged 18 and older. Among individuals between 18 and 60 years of age, our findings mirrored those of the Rural Chinese Cohort Study, demonstrating a steady increase in hypertension prevalence with age. Both our study and that of Wang et al. confirmed the relationship between WWI and hypertension frequency in individuals aged 60 and above. Our research fills a gap in the literature by uncovering the relationship between WWI and hypertension in American adults aged 18–60, revealing gender- and age-specific variations not addressed in previous studies. To explore these differences, a comparative analysis with prior studies was conducted. Our study included a larger sample of individuals over 60 years old compared to the Rural Chinese Cohort Study, which had only 650 participants with hypertension and lacked health data for those over 75. The limited number of older patients with hypertension in their sample may have contributed to a lack of statistical power in detecting the WWI-hypertension association. In contrast, Wang et al.'s study included only participants aged over 60, whereas our study population comprised U.S. adults aged 18–60, allowing us to identify patterns in younger adults.

In our research, the correlation between WWI and hypertension prevalence was particularly robust in men under 60. Higher WWI was strongly linked to an increased risk of hypertension in younger males, suggesting that WWI may be a superior predictor of obesity and hypertension risk in this demographic. This finding is consistent with prior research linking abdominal obesity to higher rates of hypertension (35). The discrepancy between older and younger individuals may stem from differences in fat distribution. Amdanee N et al. (36) investigated the impact of age on fat distribution in 102 men aged 31–83, revealing a negative correlation between age and subcutaneous and visceral fat accumulation. However, there remains a lack of large-scale studies to validate this observation, highlighting the need for further research. Regarding gender, two key factors may explain the variation in the relationship between WWI and hypertension prevalence. First, fat distribution differs between males and females; men tend to store excess fat in the abdominal region, whereas women often accumulate fat in the buttocks, resulting in a pear-shaped body (37, 38). Second, hormonal factors play a crucial role in the development of hypertension. Previous studies have shown that women generally exhibit higher levels of leptin and greater expression of leptin receptors than men, suggesting that females may release more leptin per unit of fat mass (39). Additionally, sex hormone-mediated regulation of angiotensin-converting enzyme 2 in specific tissues may contribute to the observed gender disparity in obesity-related hypertension (40).

The mechanism underlying the association between WWI and hypertension prevalence likely involves the accumulation of visceral fat. Higher WWI corresponds to greater visceral fat content, indicating excessive fat storage and reduced muscle mass (41). Excess visceral fat can contribute to systemic inflammation and activate neurohumoral pathways, which in turn elevate blood pressure and promote the development of hypertension (42, 43).

Our study offers several advantages. First, previous research involved a limited number of participants (20), while our study significantly expanded the sample size to 37,299 individuals. This larger sample better represents the U.S. population and enhances the generalizability of the findings regarding WWI. Second, prior studies focused exclusively on participants over 60 years of age, whereas our research includes a broader age range, allowing the results to be applicable to the entire U.S. adult population. Additionally, our subgroup analysis revealed variations in the relationship between WWI and hypertension based on age and gender, with WWI being a stronger predictor of hypertension prevalence in younger men—an observation not previously reported.

However, the study has several limitations. Firstly, as a cross-sectional study, it cannot establish a direct causal correlation between WWI and hypertension. Further longitudinal cohort studies are needed to confirm these findings. Secondly, although as many covariates as possible were adjusted for, hypertension is influenced by numerous factors, some of which were not captured in the NHANES database, leaving potential confounders unaccounted for. Thirdly, some data relied on participants’ self-reports, which may introduce recall bias. Lastly, the diagnosis of hypertension in NHANES was based on the average of several blood pressure readings during a single visit, differing from standard diagnostic criteria, which could have led to an overestimation of hypertension prevalence.

## 5 Conclusion

In conclusion, elevated WWI levels were strongly associated with an increased risk of hypertension, with this relationship being particularly significant in males aged 60 years or younger.

## Data Availability

Publicly available datasets were analyzed in this study. This data can be found here: https://www.jianguoyun.com/p/DUH4eb0Q2vOFDRik_94FIAA.

## References

[1] Yano Y Kim H Lee H Azahar N Ahmed S Kitaoka K Isolated diastolic hypertension and risk of cardiovascular disease: controversies in hypertension—pro side of the argument. Hypertension. (2022) 79(8):1563–70. 10.1161/HYPERTENSIONAHA.122.18459 35861749

[2] Zhou B Perel P Mensah GA Ezzati M. Global epidemiology, health burden and effective interventions for elevated blood pressure and hypertension. Nat Rev Cardiol. (2021) 18(11):785–802. 10.1038/s41569-021-00559-8 34050340 PMC8162166

[3] Roth G Mensah G Johnson C Addolorato G Ammirati E Baddour L Global burden of cardiovascular diseases and risk factors, 1990–2019: update from the GBD 2019 study. J Am Coll Cardiol. (2020) 76(25):2982–3021. 10.1016/j.jacc.2020.11.010 33309175 PMC7755038

[4] Mills K Stefanescu A He J. The global epidemiology of hypertension. Nat Rev Nephrol. (2020) 16(4):223–37. 10.1038/s41581-019-0244-2 32024986 PMC7998524

[5] Singh G Danaei G Farzadfar F Stevens G Woodward M Wormser D The age-specific quantitative effects of metabolic risk factors on cardiovascular diseases and diabetes: a pooled analysis. PLoS One. (2013) 8(7):e65174. 10.1371/journal.pone.0065174 23935815 PMC3728292

[6] Al-Makki A DiPette D Whelton P Murad M Mustafa R Acharya S Hypertension pharmacological treatment in adults: a world health organization guideline executive summary. Hypertension. (2022) 79(1):293–301. 10.1161/HYPERTENSIONAHA.121.18192 34775787 PMC8654104

[7] Mai H Li C Chen K Wu Z Liang X Wang Y Hypertension subtypes, mortality risk, and differential effects between two hypertension guidelines. Front Med (Lausanne). (2022) 9:814215. 10.3389/fmed.2022.814215 35865177 PMC9295617

[8] NCD Risk Factor Collaboration. Worldwide trends in hypertension prevalence and progress in treatment and control from 1990 to 2019: a pooled analysis of 1201 population-representative studies with 104 million participants. Lancet. (2021) 398(10304):957–80. 10.1016/S0140-6736(21)01330-1 34450083 PMC8446938

[9] Gerdts E Sudano I Brouwers S Borghi C Bruno RM Ceconi C Sex differences in arterial hypertension. Eur Heart J. (2022) 43(46):4777–88. 10.1093/eurheartj/ehac470 36136303 PMC9726450

[10] Scarpa J Bruzelius E Doupe P Le M Faghmous J Baum A. Assessment of risk of harm associated with intensive blood pressure management among patients with hypertension who smoke: a secondary analysis of the systolic blood pressure intervention trial. JAMA Netw Open. (2019) 2(3):e190005. 10.1001/jamanetworkopen.2019.0005 30848803 PMC6484649

[11] Liu W Yang C Lei F Huang X Cai J Chen S Major lipids and lipoprotein levels and risk of blood pressure elevation: a Mendelian randomisation study. EBioMedicine. (2024) 100:104964. 10.1016/j.ebiom.2023.104964 38181703 PMC10789600

[12] Hui H Kang L Min L Ai Y Ka C Louise W The impact of medium-chain triglycerides on weight loss and metabolic health in individuals with overweight or obesity: a systematic review and meta-analysis. Clin Nutr. (2024) 43(8):1755–68. 10.1016/j.clnu.2024.06.016 38936302

[13] Jayedi A Rashidy-Pour A Khorshidi M Shab-Bidar S. Body mass index, abdominal adiposity, weight gain and risk of developing hypertension: a systematic review and dose-response meta-analysis of more than 2.3 million participants. Obes Rev. (2018) 19(5):654–67. 10.1111/obr.12656 29334692

[14] Foti K Hardy S Chang A Selvin E Coresh J Muntner P. BMI and blood pressure control among United States adults with hypertension. J Hypertens. (2022) 40(4):741–8. 10.1097/HJH.0000000000003072 35001034 PMC8897212

[15] Tang N Ma J Tao R Chen Z Yang Y He Q The effects of the interaction between BMI and dyslipidemia on hypertension in adults. Sci Rep. (2022) 12(1):927. 10.1038/s41598-022-04968-8 35042940 PMC8766602

[16] Suzuki Y Kaneko H Yano Y Okada A Hashimoto Y Itoh H Threshold of BMI for the development of hypertension among Japanese adults. J Nutr. (2022) 152(11):2565–71. 10.1093/jn/nxac192 36774122

[17] Judith N Bianca S Frans K Diederick G Johanna G. Influence of weight reduction on blood pressure: a meta-analysis of randomized controlled trials. Hypertension. (2003) 42(5):878–84. 10.1161/01.HYP.0000094221.86888.AE 12975389

[18] David A Dana T Robert K Anita C. Benefits and risks of bariatric surgery in adults: a review. JAMA. (2020) 324(9):879–87. 10.1001/jama.2020.12567 32870301

[19] Nimptsch K Konigorski S Pischon T. Diagnosis of obesity and use of obesity biomarkers in science and clinical medicine. Metab Clin Exp. (2019) 92:61–70. 10.1016/j.metabol.2018.12.006 30586573

[20] Yusuf S Hawken S Ounpuu S Bautista L Franzosi M Commerford P Obesity and the risk of myocardial infarction in 27,000 participants from 52 countries: a case-control study. Lancet. (2005) 366(9497):1640–9. 10.1016/S0140-6736(05)67663-5 16271645

[21] Park Y Kim N Kwon T Kim S. A novel adiposity index as an integrated predictor of cardiometabolic disease morbidity and mortality. Sci Rep. (2018) 8(1):16753. 10.1038/s41598-018-35073-4 30425288 PMC6233180

[22] Hao H Ping N Si Z Xiao J Ming Z Wan M The association of body mass index and weight waist adjustment index with serum ferritin in a national study of US adults. Eur J Med Res. (2023) 28(1):374. 10.1186/s40001-023-01343-9 37749647 PMC10521392

[23] Jia W Jin G. Is weight-adjusted waist index more strongly associated with diabetes than body mass index and waist circumference? Results from the database large community sample study. PLoS One. (2024) 19(9):e0309150. 10.1371/journal.pone.0309150 39325793 PMC11426486

[24] Kim J Choi J Vella C Criqui M Allison M Kim N. Associations between weight-adjusted waist index and abdominal fat and muscle mass: multi-ethnic study of atherosclerosis. Diabetes Metab J. (2022) 46(5):747–55. 10.4093/dmj.2021.0294 35350091 PMC9532169

[25] Ding C Shi Y Li J Li M Hu L Rao J Association of weight-adjusted-waist index with all-cause and cardiovascular mortality in China: a prospective cohort study. Nutr Metab Cardiovasc Dis. (2022) 32(5):1210–7. 10.1016/j.numecd.2022.01.033 35277327

[26] Cai S Zhou L Zhang Y Cheng B Zhang A Sun J Association of the weight-adjusted-waist index with risk of all-cause mortality: a 10-year follow-up study. Front Nutr. (2022) 9:894686. 10.3389/fnut.2022.894686 35694172 PMC9174751

[27] Zai T Peng Z Gen M. Association of weight-adjusted waist index with cardiovascular disease and mortality among metabolic syndrome population. Sci Rep. (2024) 14(1):18684. 10.1038/s41598-024-69486-1 39134613 PMC11319818

[28] Li Q Qie R Qin P Zhang D Guo C Zhou Q Association of weight-adjusted-waist index with incident hypertension: the rural Chinese cohort study. Nutr Metab Cardiovasc Dis. (2020) 30(10):1732–41. 10.1016/j.numecd.2020.05.033 32624344

[29] Wang J Yang Q Chai D Su Y Jin Q Wang J. The relationship between obesity associated weight-adjusted waist index and the prevalence of hypertension in US adults aged ≥60 years: a brief report. Front Public Health. (2023) 11:1210669. 10.3389/fpubh.2023.1210669 37869197 PMC10587597

[30] Speer H D'Cunha N Naumovski N McKune A. Sex, age, BMI, and C-reactive protein impact the odds of developing hypertension-findings based on data from the health and retirement study (HRS). Am J Hypertens. (2021) 34(10):1057–63. 10.1093/ajh/hpab088 34106249

[31] Muhammad T Paul R Rashmi R Srivastava S. Examining sex disparity in the association of waist circumference, waist-hip ratio and BMI with hypertension among older adults in India. Sci Rep. (2022) 12(1):13117. 10.1038/s41598-022-17518-z 35907951 PMC9338983

[32] Krumholz HM de Lemos JA Sattar N Linetzky B Sharma P Mast CJ Tirzepatide and blood pressure reduction: stratified analyses of the SURMOUNT-1 randomised controlled trial. Heart. (2024) 110(19):1165–71. 10.1136/heartjnl-2024-324170 39084707 PMC11420724

[33] Ahima R Lazar M. Physiology. The health risk of obesity–better metrics imperative. Science. (2013) 341(6148):856–8. 10.1126/science.1241244 23970691

[34] Liu S Yu J Wang L Zhang X Wang F Zhu Y. Weight-adjusted waist index as a practical predictor for diabetes, cardiovascular disease, and non-accidental mortality risk. Nutr Metab Cardiovasc Dis. (2024) 34(11):2498–510. 10.1016/j.numecd.2024.06.012 39117486

[35] Ostchega Y Hughes J Terry A Fakhouri T Miller I. Abdominal obesity, body mass index, and hypertension in US adults: NHANES 2007–2010. Am J Hypertens. (2012) 25(12):1271–8. 10.1038/ajh.2012.120 22895451 PMC9745582

[36] Amdanee N Di W Liu J Yu J Sheng Y Lv S Age-associated changes of resting energy expenditure, body composition and fat distribution in Chinese Han males. Physiol Rep. (2018) 6(23):e13940. 10.14814/phy2.13940 30536574 PMC6286433

[37] Dixon A Peters U. The effect of obesity on lung function. Expert Rev Respir Med. (2018) 12(9):755–67. 10.1080/17476348.2018.1506331 30056777 PMC6311385

[38] Tchernof A Brochu D Maltais-Payette I Mansour M Marchand G Carreau A Androgens and the regulation of adiposity and body fat distribution in humans. Compr Physiol. (2018) 8(4):1253–90. 10.1002/cphy.c170009 30215860

[39] Faulkner JL Belin de Chantemèle EJ. Sex differences in mechanisms of hypertension associated with obesity. Hypertension. (2018) 71(1):15–21. 10.1161/HYPERTENSIONAHA.117.09980 29133358 PMC5730468

[40] Kaneva A Bojko R. Sex differences in the association between obesity and hypertension. Arch Physiol Biochem. (2023) 129(3):682–9. 10.1080/13813455.2020.1861027 33476209

[41] Kim N Park Y Kim N Kim S. Weight-adjusted waist index reflects fat and muscle mass in the opposite direction in older adults. Age Ageing. (2021) 50(3):780–6. 10.1093/ageing/afaa208 33035293

[42] Hall M Cohen J Ard J Egan B Hall J Lavie C Weight-loss strategies for prevention and treatment of hypertension: a scientific statement from the American Heart Association. Hypertension. (2021) 78(5):e38–50. 10.1161/HYP.0000000000000202 34538096

[43] Pierce G Coutinho T DuBose L Donato A. Is it good to have a stiff aorta with aging? Causes and consequences. Physiology (Bethesda). (2022) 37(3):154–73. 10.1152/physiol.00035.2021 34779281 PMC8977146

