# Supplementary material for: Correlation between weight-adjusted-waist index and hypertension in the US population: based on data from NHANES 2005–2018
Source: Front Cardiovasc Med. 2024 Nov 12;11:1416836. doi: 10.3389/fcvm.2024.1416836 (PMC11588733; doi:10.3389/fcvm.2024.1416836)
Supplement: Supplementary file 1 [file Datasheet1.pdf]

Supplementary Tables

Table S1 Pearson correlation analysis of correlation between WWI and BMI

	r(95%CI)	P
Gender		
Male	0.56(0.55-0.58)	<0.0001
Female	0.49(0.48-0.51)	<0.0001
Age		
≤60	0.58(0.57-0.59)	<0.0001
>60	0.41(0.40-0.43)	<0.0001
Smoking		
Yes	0.52(0.51-0.53)	<0.0001
No	0.51(0.50-0.52)	<0.0001

Table S2 Association between body mass index and the prevalence of hypertension

body mass index	Model 1		Model 2		Model 3	
	OR (95%CI)	P-value	OR (95%CI)	P-value	OR (95%CI)	P-value
Continuous	1.07(1.07,1.08)	<0.0001	1.08(1.08,1.09)	<0.0001	1.06(1.06,1.07)	<0.0001

Model 1:Unadjusted;Model 2: Adjusted for gender, age, race at baseline;Model 3: Adjusted for gender, age, race, education level, smoking status, Fasting plasma glucose, Hemoglobin A1c, Serum creatinine, Serum uric acid, Total cholesterol, fasting plasma glucose, total cholesterol, high-density lipoprotein cholesterol,low-density lipoprotein cholesterol,triglyceride,alanine transaminase, aspartate transaminase at baseline.
